# Supplementary material for: The interactome of intact mitochondria by cross-linking mass spectrometry provides evidence for coexisting respiratory supercomplexes
Source: Mol Cell Proteomics. 2017 Dec 8;17(2):216–32. doi: 10.1074/mcp.RA117.000470 (PMC5795388; doi:10.1074/mcp.RA117.000470)
Supplement: Supplemental Data [file supp_RA117.000470_133922_0_supp_23978_fzffwf.zip › spectra_annotation/mito_spectra_annotation/87-1-2-1-9-1.pdf]

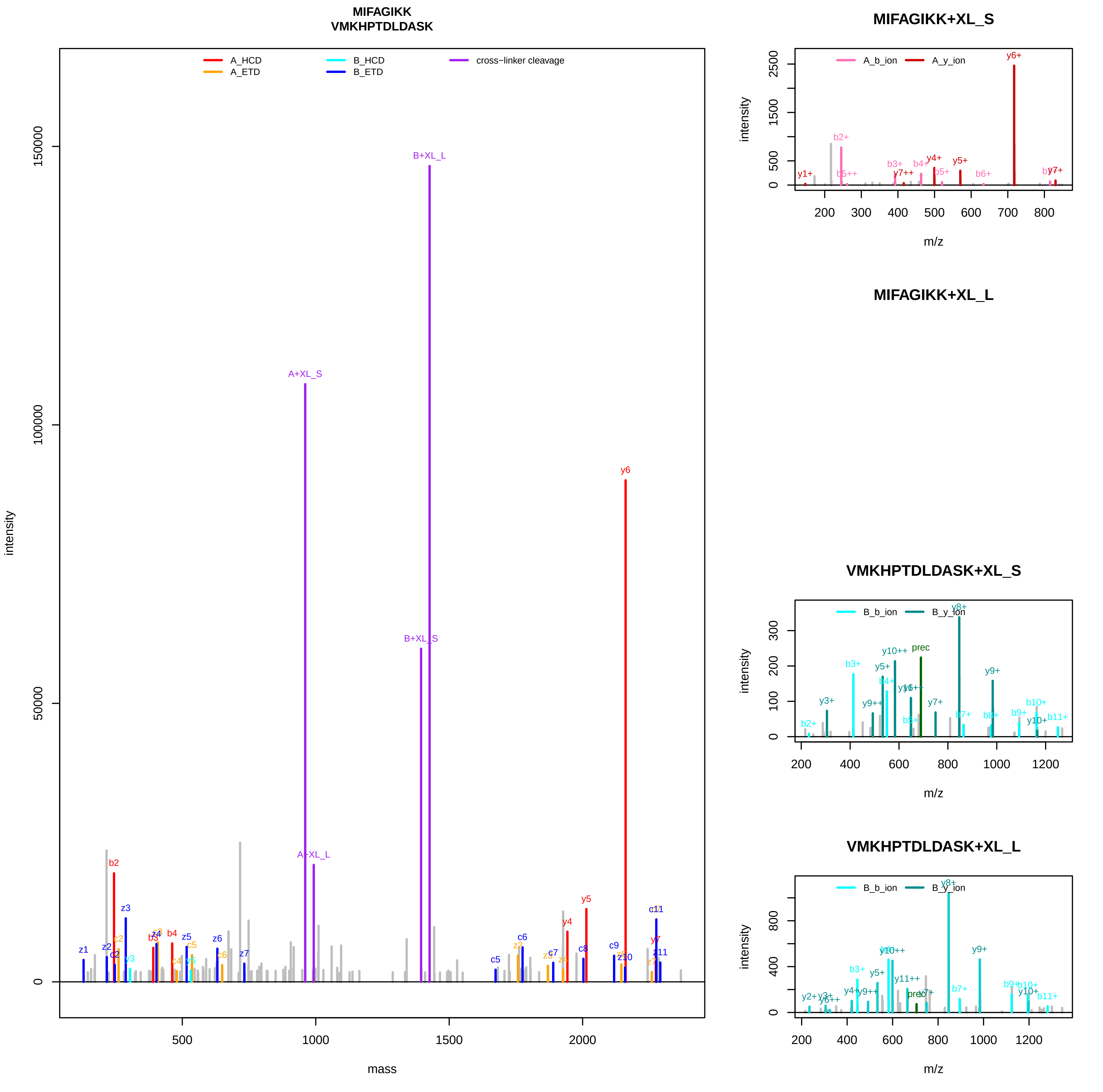

MIFAGIKK
VMKHPTDLDASK
A_HCD
A_ETD
B_HCD
B_ETD
cross−linker cleavage
intensity
mass
0
50000
100000
150000
500
1000
1500
2000
B+XL_L
A+XL_S
B+XL_S
A+XL_L
y6
b2
z3
b4
z5
z6
z7
c5
c6
c7
c8
c9
z10
c11
z11
y4
y5
z1
z2
c2
MIFAGIKK+XL_S
A_b_ion
A_y_ion
m/z
y6+
b2+
y4+
y5+
b3+
b4+
y7++
b5+
b6+
b5++
y1+
y7+
MIFAGIKK+XL_L
VMKHPTDLDASK+XL_S
B_b_ion
B_y_ion
y8+
prec
y10++
b3+
y5+
y9+
y3+
y9++
y7+
b10+
b9+
y10+
b11+
b2+
b7+
b8+
VMKHPTDLDASK+XL_L
y11++
y2+
y6++
y4+
